# Role of global public sector research in discovering new drugs and vaccines

**DOI:** 10.1007/s10961-023-10007-z

**Published:** 2023-04-27

**Authors:** Ashley J. Stevens, David E. Benson, Sara E. Dodson, Jonathan J. Jensen, Mark L. Rohrbaugh

**Affiliations:** 1 Focus IP Group, LLC, 70 Yale Street, Winchester, MA 01890 USA; 2 Department of Strategic Management and Operations, Utah Valley University, Orem, UT USA; 3 Office of Science Policy, National Institutes of Health, Bethesda, MD USA; 4 Office of Technology Development, Salk Institute for Biological Studies, La Jolla, CA USA

**Keywords:** Drugs, Research, Public sector, Global, Innovation, O34 intellectual property, Intellectual capital

## Abstract

Analysis of international public-sector contributions to Food and Drug Administration (FDA)-approved drugs and vaccines allows for a more thorough examination of the global biomedical innovation ecosystem by institution of origin. Using new and existing methods, we have identified 364 FDA-approved drugs and vaccines approved from 1973 to 2016 discovered in whole or in part by Public Sector Research Institutions (PSRIs) worldwide. We identified product-specific intellectual property contributions to FDA-approved small molecule and biologic drugs and vaccines from the FDA Orange Book, our peer network, published studies, and three new sources: reports of medical product manufacturers’ payments to physicians and teaching hospitals under The Sunshine Act of 2010, a paper by Kneller and 64 royalty monetization transactions by academic institutions and/or their faculty that one of us (AS) maintains. We include a total of 293 drugs discovered either wholly by a US PSRI or jointly by a U.S. and a non-U.S. PSRI. 119 FDA-approved drugs and vaccines were discovered by PSRIs outside the U.S. Of these, 71 were solely discovered outside the US, while 48 also involved intellectual property contributions by US PSRIs. In the context of the global public sector landscape, the US dominates drug discovery, accounting for two-thirds of these drugs and many of the important, innovative vaccines introduced over the past 30 years. Contributions by Canada, UK, Germany, Belgium, Japan, and others each amount to 5.4% or less of the total.

**Supplementary Information:**

The online version contains supplementary material available at 10.1007/s10961-023-10007-z.

## Introduction

The critical role of academic research in providing the scientific underpinning for new drug discovery is undisputed.

Cleary et al. (2018)quantified the cost of this contribution by examining the publication base underlying the 210 new molecular entities (NMEs) approved by the FDA 2010–2016 and 151 biological targets associated with these NME’s. 131,092 publications were associated with the NME’s and 1,966,281 publications with the biological targets. Publications acknowledging NIH funding (29% of the total) represented funding of over $100 billion in inflation adjusted terms between 1985 and 2016. Over $64 billion of this funding was associated with 84 first-in-class NME’s during this period binding to 77 molecular targets. The earliest publications related to the targets, including 96% of those with NIH funding, were published up to 30 years prior to drug approval, while those related to the NME’s were published much closer to drug approval.

However, our interest was not in the scientific contribution of public sector research institutions (PSRIs) to the discovery of new drugs, but rather to identify PSRIs’ contribution to the intellectual property (IP) created in the discovery of new drugs, specifically, IP required to make, use or sell the drug.

Historically, it was generally believed that the applied research to translate basic scientific discoveries on, say, a newly discovered drug targets into a new drug was carried out solely in the private sector. However, a number of publications have confirmed an important PSRI role in drug discovery with widely different findings. Kaitin et al. (1993) found that 7.6% of the new drugs approved 1981–1990 did not originate in the pharmaceutical industry. DiMasi et al. (2003) found that of 284 new drugs approved in the U.S. 1990–1999, 6.7% percent did not originate from industrial sources. Zycher et al. (2010) found that of 35 drugs, only one originated from PSRIs.

Sampat and Lichtenberg (2011) looked at 478 New Molecular Entities (NME’s) approved between 1988 and 2005 and obtained patent data on 379 of them. They found that PSRI-owned patents were associated with 9% of all 379 drugs, with 3.1% of standard review drugs and 17.4% of priority review drugs having PSRI-owned patents, consistent with our 2011 paper.

Kneller (2010) identified the inventors of 252 drugs approved from 1998 to 2007 and their type of institutional affiliation by country and concluded that inventors at biotech companies and universities accounted for 23% and 30%, respectively, of the scientifically innovative drugs and half of those that addressed unmet medical needs, but a somewhat lower percentage of all new drug approvals. Kneller’s paper was published after the submission of our 2011 paper and, as discussed below, was one of our four primary sources for this work.

Lincker et al. (2014) looked at 94 new active substances (NMEs in U.S. terminology) approved for marketing in the EU 2010–2012. They found that 16 originated in academic, public bodies or public–private-partnerships but did not identify those originators by name.

Stevens et al. (2011) identified 293 drugs discovered in whole or in part by US PSRIs.

## Methods

We use the term PSRI broadly to include universities, research hospitals, not-for-profit research institutes and government laboratories world-wide which contributed to products approved by the Food and Drug Administration (FDA) in the U.S.^1^ We included eight drugs invented by physicians in private practice in the U.S., generally new uses for existing drugs.

We define “drug” as any product that received U.S. marketing approval from either the Center for Drug Evaluation and Research (CDER) or the Center for Biologics Evaluation and Research (CBER) of the FDA.^2^ This includes small molecule drugs (including over-the-counter (OTC) drugs), protein-based biologic drugs, vaccines and in vivo diagnostics. We did not include serum-derived biologic drugs. We did not include insulin, warfarin, l-DOPA or the early vaccines and antibiotics discovered in PSRIs pre- and immediately post-WWII.

We included drugs receiving FDA approval up to December 31, 2016.

For inclusion, a PSRI needed to have created, product-specific intellectual property (IP), such as patented inventions or proprietary materials required to make, use or sell the drug, and to have transferred rights to that IP to a company through a license. The licensed IP was generally a patent covering the drug ingredient, its formulation or use, though a few transactions involved know-how such as proprietary biological materials or data pertaining to regulatory approval. The extent of the PSRI IP contribution to an individual drug’s total IP protection ranged from the discovery of and patent on the drug’s target to all of the Orange Book listed patents for the product being owned by the PSRI.

As in our earlier paper, we excluded the role of PSRIs in developing platform technologies that contributed to the development of whole new classes of biological drugs. While these platform technologies enabled the development of substantial numbers of products—e.g., all antibody drugs required a license to the Cabilly patent for their manufacture until it expired in 2018—the PSRI scientists who invented these platforms generally did not use them to develop specific drug candidates.

A broad range of relationships is encompassed in our study. In most cases, the PSRI discovered the composition-of-matter or a new use for an existing compound in the course of grant-funded research and subsequently licensed the IP to a company that developed the drug. Some drugs resulted from public–private partnerships. Some products utilize patents from multiple PSRIs. Sometimes simultaneous inventions in the public and private sectors resulted in interferences which were resolved through negotiation, with the parties agreeing to choose the strongest patent and share rights in the invention. Other academic drug discoveries seem to have made their way “out the back door,” with the academic discoverers assigning their interest to a company rather than to the academic institution where they carried out the work. And, finally, in a few cases, the developing company disputed the PSRI’s IP, resulting in litigation and a license being imposed judicially.

It is challenging to determine which drugs were discovered at PSRIs—in their public announcements companies have an incentive to stress their internal discovery capabilities rather than emphasizing that they licensed a drug from a PSRI that had originally discovered it, though they are generally required to disclose the PSRI licenses they have signed in their filings with the Securities and Exchange Commission (SEC).

As in our earlier work, our primary source was the FDA’s Orange Book which contains details of the patent protection underlying small molecule drugs approved under NDA’s, but not Biologics License Applications (BLA’s), which cover therapeutic biologics and vaccines. One issue is that when a patent listed in the Orange Book expires it is no longer included in the Orange Book. Sampat has created a comprehensive database of all products that have ever had patents listed in the Orange Book by obtaining printed copies of older Orange Books and hand coding the information (Sampat, 2009). He has kept this listing current and generously made this data available to us. In total, 171 drugs included U.S. and non-U.S. PSRI patents in their Orange Book listings. If a patent assigned to a PSRI was listed in the Orange Book we accepted it as prima facia evidence of a PSRI role in the drug’s discovery, even if we could not identify the transactions transferring rights to the IP, which was the case for some drugs discovered in the 1960s and ‘70’s.

Another source was the research of Kneller (2010), which attributed 83 drugs to co-discovery by PSRIs. We did extensive research to confirm that the drugs he identified met our criteria of IP being created and licensed by the PSRI. We were not able to confirm that fourteen of these drugs met our criteria. In a further seven, we could not verify that the PSRIs met our criteria—in some cases, there was no IP created by the individuals Kneller identified, or the IP had expired by the time development commenced for another indication, or we could not identify any transactions transferring the IP. Overall, we included 66 drugs identified by Kneller, of which 40 were not listed in the Orange Book.

We used a new source, the Sunshine Act^3^ implemented in 2010. It requires medical suppliers—pharmaceutical, biopharmaceutical and device companies—to report payments over $10 to any physician or hospital, categorized into one of fifteen categories, including “License and Royalties.” Payment of substantial amounts in this category is evidence of an IP transaction. To be included in our study, we applied a cutoff that the physician had received at least $100,000 over a three-year period. The Sunshine Act allowed us to identify physicians who own IP on approved drugs that had been licensed to a drug company which paid them royalties and we identified the IP using the USPTO database. The limitation of the Act is that it does not include payments to universities, as opposed to their teaching hospitals, nor to Ph.D.’s. This revealed 31 drugs as coming from PSRIs and two invented by physicians in private practice, of which 19 were not identified by Kneller or the Orange Book.

Our fourth major source was a compilation of 64 royalty monetization transactions by academic institutions and/or their faculty that one of us (AS) maintains. Only the owners of the IP (and participating inventors) receive royalties that can be monetized. This source identified 52 drugs, of which 16 were not revealed by the previous three sources. We used this information to identify the inventors and the IP that was licensed.

These four major sources accounted for a total of 246 drugs or 67% of the total we identified; the remaining 118 (33%) were identified by sources such as press announcements, litigation, conversations with colleagues, etc. The “back door” pathways discussed above were identified through these discussions and verified via identification of academic inventors who assigned their interest in one or more patents to a corporate entity and not their employing PSRI. We therefore believe that our research provides the most complete compilation of drugs owing their origins to PSRIs.

We obtained additional information from the USPTO database, federal grant funding databases, the FDA’s drug^4^ and biologic^5^ approval databases and the Cortellis database offered by Clarivate Analytics^6^ to trace the various transactions during the drugs’ development from discovery to market. In this way, we were able to identify in detail the timeline of the drug’s progress from bench to bedside, its approved indication(s) and the build-up in value in the various transactions along the way. We have reviewed this data as it applies to the drugs in our 2011 paper (Stevens, 2015).

## Results

### Number of products

We have identified a further 140 FDA approved drugs that were discovered at least in part by U.S. PSRIs in addition to the 153 we reported in 2011, for a total of 293. Of the newly identified drugs, 83 received FDA approval after the August 31, 2009 cutoff for our 2011 study, while 57 were approved earlier, but their PSRI origins have only now been identified.

In addition, we have identified 119 FDA approved drugs that were discovered in whole or in part by PSRIs outside the U.S. Of these, 71 were solely discovered by PSRIs outside the U.S., while 48 also involved IP contributions by U.S. PSRIs and so are included in the 293 figure above.

We excluded bimatoprost, because the drug was discovered at Procter & Gamble who donated the patents to Duke University, which made no IP contribution to the commercialized drug, but we included lomitapide, because the University of Pennsylvania obtained its own Orange Book-listed patents on the method of treatment after the patents on the drug were donated to Penn by Bristol-Myers.

In total, therefore, we have identified 364 FDA approved drugs discovered in whole or in part by PSRIs worldwide.

The oldest drug included in our study is silver sulfaziadine, invented at Columbia in 1967 and approved in December 1973.

### Types of products

The distribution of the 364 products between the four broad categories of therapeutic products is shown in Table 1. Particularly noteworthy are the 23 vaccines. Nearly all important, innovative vaccines introduced over the past 30 years were invented by PSRIs, a trend continuing up to the response to the current pandemic. Vaccines are expensive to manufacture and uptake by otherwise healthy people is often unpredictable, resulting in reduced incentives for vaccine development by corporations compared to drugs. Our findings of significant intellectual property contributions by PSRIs to vaccines for infectious diseases are consistent with the lower level of risk tolerated by commercial vaccine developers (Plotkin, 2017; Rappuoli, 2019).

**Table 1:** Types of products

Type of product	Number
New chemical entity	234
Biologic	78
Vaccine	23
Over the counter	2
NCE/OTC	25
In-vivo diagnostic	2
Total	364

### Therapeutic categories

The therapeutic categories of the 364 products are shown in Table 2. It shows that PSRI’s have discovered drugs across a broad spectrum of therapeutic categories. Oncology and Infectious Diseases account for 43% of the total. The pharmaceutical industry does not assign such a high priority to infectious diseases and a number of major pharmaceutical companies have terminated their infectious disease drug discovery efforts, so relatively high NIH funding, resulting in a significant PSRI effort in this area, is an important contribution to public health. We aligned the therapeutic categories in Table 2 with the corresponding disease-specific institute of the NIH and found that the total number of drugs generally correlates with the 2016–2018 budgets of those institutes;^7^ however, we did not investigate the source of funding for these inventions. NIH funding and Institute scope are in the purview of Congress and reflect the priorities and advocacy of patients and industry.

**Table 2:** Therapeutic categories of products

Therapeutic area	Number	
Oncology	88	24.2%
Infectious disease	68	18.7%
Metabolic	52	14.3%
CNS	45	12.4%
Cardiology	22	6.0%
Renal	14	3.8%
Dermatology	13	3.6%
Gastroenterology	12	3.3%
Women’s Health	12	3.3%
Ophthalmology	9	2.5%
Immunology	7	1.9%
Anesthesiology	6	1.6%
Pulmonary	5	1.4%
Urology	4	1.1%
Allergy	2	0.5%
Dental	2	0.5%
Emergency Medicine	2	0.5%
Otolaryngology	1	0.3%
Total	364	

### Discovering countries

Table 3 shows 444 drug discoveries by country, which exceeds the 364 unique drugs discovered by all PSRIs, because scientists in different countries frequently collaborate and develop jointly-owned IP, and different institutions sometimes develop IP covering different aspects of a drug, e.g., active ingredient, formulation, and method of treatment or delivery. Most PSRI discoveries were in N. America and Europe, with smaller contributions from the Asia–Pacific region and the Middle East. U.S. institutions discovered or co-discovered the most drugs (293), followed by Canada, U.K., Germany, and Belgium.

**Table 3:** Discovering regions and countries

Region/country	No. of drugs		Academic R&D Expend 2010–2016 ($mm)	Drug/$billion Academic R&D	vs US (%)
*N. America*					
US	293	66.0%	529,880.0	0.55	100
Canada	24	5.4%	86,585.2	0.28	50
Subtotal	317	71.4%			
*Europe*					
Germany	21	4.7%	149,176.5	0.14	25
UK	21	4.7%	92,384.2	0.23	41
Belgium	15	3.4%	19,368.8	0.77	140
Czech Republic	12	2.7%	10,381.4	1.16	209
France	8	1.8%	101,228.2	0.08	14
Sweden	4	0.9%	31,420.9	0.13	23
Holland	2	0.5%	43,551.9	0.05	8
Switzerland	1	0.2%	30,566.2	0.03	6
Russia	1	0.2%	27,477.0	0.04	7
Subtotal	85	19.1%			
*Asia–Pacific*					
Australia	14	3.2%	52,815.1	0.27	48
Japan	14	3.2%	171,056.5	0.08	15
China	2	0.5%	187,484.7	0.01	2
Subtotal	30	6.8%			
*Middle East*					
Israel	12	2.7%	11,368.8	1.06	191
Total	444				

### Discovering institutions

165 PSRIs and individuals (as a group) have discovered or co-discovered the 364 drugs. Supplementary Table 2 lists all 165 PSRIs. Table 4 shows the 14 organizations that discovered or co-discovered eight or more drugs. Six PSRIs outside the U.S. are on this list.

**Table 4:** Top discovering public sector research institutions

Public Sector Research Institutions	Number discovered
National Institutes of Health	27
U. of California	21
Emory University	18
U. of British Columbia	16
K.U. Leuven	14
Tufts Medical Center	13
Czech Academy of Sciences	12
Hans Knoell Institute	12
Tufts University	12
U. of Toronto	12
Massachusetts General Hospital	9
Memorial Sloan Kettering	9
U. of Texas	9
Columbia University	8
Individuals	8
Weizmann Institute of Science	8

Teaching hospitals are generally independent corporations and so they, rather than the university with which they are affiliated, own the IP their researchers create, so the hospitals, rather than their affiliated universities, are included in Supplementary Table 2 and also in Table 4.

Two qualifiers to these results should be noted:

Czech Academy of Sciences, Emory, Institute Armand Frappier, K.U. Leuven, McGill, U. of Minnesota and Yale discovered one or more anti-virals, particularly anti-retrovirals, and some of these NCE’s were formulated into multiple Fixed Dose Combination (FDCs) which each required an NDA, resulting in high numbers of NDAs from a relatively small number of NCEs.

Two international collaborations—between Tufts Medical Center/Tufts University/University of Toronto and between the Hans-Knoell-Institute/University of British Columbia—discovered the use of dipeptidyl peptidase-IV inhibitors to treat type 2 diabetes. The IP was licensed non-exclusively to four companies, resulting in 12 NDAs, which accounts for these institution’s high rankings.

### Rate of discovery

**Fig. 1:**
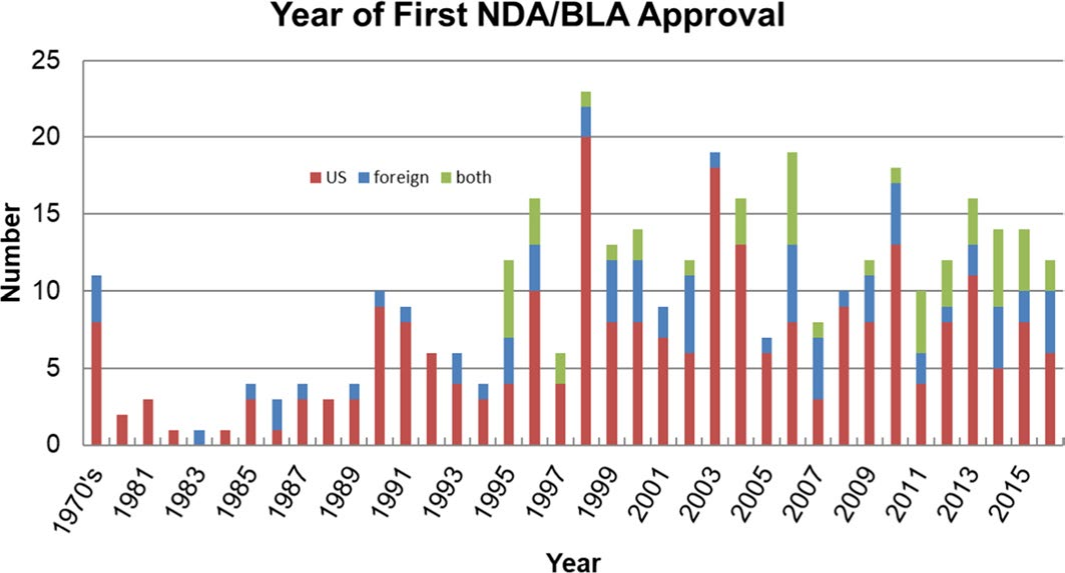
Year of approval of first NDAs and BLAs

Figure 1 shows the number of first NDA/BLA approvals each year for drugs in this study, identified by their geographic origins. It shows that approvals started to climb in 1995 and shows that there has been a steady flow of new PSRI-discovered drug approvals each year since. We found a median period of 12.51 years from discovery to FDA approval, and that the rate of discovery of new drugs started to increase in the early 1980s.

The contribution of PSRIs outside the US appears to have been increasing in recent years.

## Discussion

Our 2011 paper showed that PSRIs in the US make an important, and previously under-appreciated, contribution to the discovery of new drugs. In this paper, we extend our research to show that PSRIs outside the US also make significant contributions to new drug discovery and, for the first time, identify those institutions and the drugs they have contributed.

That said, U.S. PSRIs dominate academic drug discovery, having discovered two-thirds of all PSRI-discovered drugs approved by the FDA. The next most prolific countries, Canada and the U.K. each discovered around 5% of the total. While most large, developed countries have discovered at least one drug, countries with well-developed academic research ecosystems, such as Italy, Spain and South Korea are absent from Table 3.

We examined the volume of PSRI research and its correlation with drug discovery in these different countries.

We use OECD data on Academic R&D spending from 2010 to 2016 (adjusted for inflation and purchasing power parity (PPP)) as a proxy for the volume of academic research in each country through the study period. In Table 3 we calculate the number of drugs discovered per billion dollars of academic R&D spending over this period. This analysis shows that while most countries convert academic R&D investment into drug discoveries at a lower rate than the U.S., three countries—Belgium, the Czech Republic, and Israel—generate significantly more drugs per billion dollars of academic R&D than the U.S. However, as noted above, the figures for Belgium and the Czech Republic predominantly reflect a highly productive collaboration between K.U. Leuven and the Czech Academy of Sciences in anti-viral research that resulted in a number of HIV FDCs. As noted above, several institutions in both the U.S. and Canada also had multiple drugs result from single discoveries. The impact of the U.S. institutions—Emory, Tufts, U. of Minnesota and Yale—on the U.S. total is minor, while the multiple approvals from the discoveries of the Institute Armand Frappier, McGill, Toronto and UBC in Canada account for a significant percentage of the Canadian total.

Although the FDA currently is not accepting clinical research from Chinese companies performed in China, we do not believe this accounts for the low total for China because promising drug discoveries would likely be partnered with multinational drug companies for late-stage development.

While there is an extensive literature on the factors that contribute to applied versus basic research in academic institutions and the proclivity of academic researchers to engage in patenting or applied activity, we believe our dataset may be inadequate for a rigorous analysis. The number of drugs we identify is too small and, as we have shown, is distorted by a few special cases to attempt to relate individual institutional or even national drug outputs to any of these factors.

## Supplementary Information

Below is the link to the electronic supplementary material.

Supplementary file1 (XLSX 45 kb)

Supplementary file2 (XLSX 12 kb)

## Footnotes

1 US Food and Drug Administration, Approved Drug Products with Therapeutic Equivalence Evaluations: Orange Book. https://www.fda.gov/drugs/drug-approvals-and-databases/approved-drug-products-therapeutic-equivalence-evaluations-orange-book Accessed October 4, 2022. The Orange Book provides a listing of patents and other regulatory exclusivity protecting approved drugs.

2 We identified some drugs that originated at both U.S. and non-U.S. institutions that only received marketing approval outside the U.S but did not include them in our totals.

3 The Affordable Care Act (ACA) of 2010, Public Law 111–148, Sect. 6002, codified at 42 USC §1320a-7 h.

4 FDA-Approved Drugs https://www.accessdata.fda.gov/scripts/cder/daf/index.cfm Accessed October 4, 2022.

5 FDA Biological Approvals by Year. https://www.fda.gov/vaccines-blood-biologics/development-approval-process-cber/biological-approvals-year Accessed October 4, 2022.

6 At various times in its history, this database has been known as the rDNA database offered by Recombinant Capital and the IQ Series by Thomson-Reuters.

7 NIH Office of Budget, Appropriations History by Institute/Center. https://officeofbudget.od.nih.gov/approp_hist.html. Accessed October 4, 2022.a.
